# Fungicide Treatments to Control Seed-borne Fungi of Sunflower Seeds

**DOI:** 10.3390/pathogens9010029

**Published:** 2019-12-27

**Authors:** Mandela Elorm Addrah, Yuanyuan Zhang, Jian Zhang, Lin Liu, Hongyou Zhou, Weidong Chen, Jun Zhao

**Affiliations:** 1 College of Horticulture and Plant Protection, Inner Mongolia Agricultural University, Hohhot 010018, China; addeahmandela@yahoo.com (M.E.A.); zhj19890128@126.com (J.Z.); liulin@emails.imau.edu.cn (L.L.); hongyouzhou2002@aliyun.com (H.Z.); 2 Institute of Grassland Research of CAAS, Huhhot 010010, China; zhangyuanyuan01@caas.cn; 3 Department of Plant Pathology, Washington State University, Pullman, WA 99164, USA; w-chen@wsu.edu

**Keywords:** flusilazole, sunflower, seed-borne fungi, seed coat contamination, seed pretreatment

## Abstract

Seed-borne fungi in 69 sunflower cultivars were evaluated which comprised 52 confectionery and 17 oilseed types. Seed coats were placed on both NP-10 (Nonylphenol Ethoxylate based surfacant −10) and potato dextrose agar (PDA) media to culture fungi. The rate of contamination among the different varieties was calculated by counting seed coats with fungal colonies. The rate of contamination in the confectionary group (88%) was significantly (*p* ≤ 0.05) higher than in the oilseed group (71%). Of the 52 confectionery varieties, the dominant fungi recovered were *Verticillium dahliae* along with *Alternaria* spp., *Fusarium* spp., and *Rhizopus* spp., whereas the oilseed type varieties were contaminated with only *V. dahliae.* Molecular identification of fungal species via BLAST (Basic Alignment Search Tool) was performed on fungal sequences obtained from PCR (Polymerase Chain Reaction) analysis. The results included five *Alternaria* spp. that included *Alternaria tenuissima*, *Alternaria alternata*, *Alternaria helianthiinficiens*, *Alternaria longipes*, and *Alternaria tamaricis*, three *Fusarium* spp. such as *Fusarium oxysporum*, *Fusarium incarnatum*, and *Fusarium proliferatum*, and *V. dahliae* and *Cladosporium cladosporioides*. These were identified from pure fungal cultures recovered from seed coats. To efficiently control seed-borne fungi, four broad spectrum fungicides (carbendazim, triadimefon, caprio F-500, and flusilazole) were screened against *V. dahliae* isolate Gn3, which was isolated from a diseased LD 5009 sunflower plant. Flusilazole was selected based on its low half-maximal effective concentration value (EC_50_), 78.7 µg/mL. Seeds of diseased LD 5009 plants obtained from two different locations treated with formulated flusilazole fungicide at optimum parameters showed a significant (*p* ≤ 0.05) increase in seed germination and a decrease in contamination rate from 98% to less than 10%. The results affirmed that confectionery cultivars are much more susceptible to fungal contamination than oilseeds, and also that seed pretreatment is a suitable way to prevent the spread of soil- and seed-borne fungi in sunflower production.

## 1. Introduction

Sunflower (*Helianthus annuus* L.) is one of the top oilseed crops grown for their edible oil. Sunflower seeds contain over 40% edible oil and 23% proteins and are a good source of fiber, vitamin E, copper, zinc, and B complex vitamins [1]. A number of soil- and seed-borne fungal pathogens and other phytopathogenic microorganisms negatively affect the sunflower cultivation and seed production. Planted seeds are mostly affected during the early stages of germination and results in poor seedling emergence [2]. Contaminated seeds develop damping-off, root rot, leaf spot, or stem rot in the field causing huge economic losses of sunflower production [3,4]. *Fusarium* spp. and *Verticillium* spp. infect plant roots and expand via the vascular system causing severe leaf wilting, stunted plant growth, and vascular discoloration [5]. A survey conducted on seed-borne fungal diseases associated with sunflower using the standard blotter method showed that *Alternaria helianthi*, *Rhizoctonia bataticola*, and *Alternaria alternata* had a percentage occurrence rate of 55%, 15%, and 9% respectively. Other fungal species, including *Penicillium* spp., *Aspergillus niger*, *Aspergillus flavus*, and *Rhizopus* spp., were also detected on seeds of sunflower; however, *V. dahliae* and *Fusarium* spp. were not recorded in this study [6]. *V. dahliae* can colonize the pericarp and testa of certain safflower seeds [7] and also the pericarp and endosperm of lettuce seed [8], hence the possibility of seeds being the main dispersal agents for long-distance transmission of *V. dahliae* [7,9,10,11,12]. *A. alternata*, *Rhizopus stolonifer*, and *Fusarium* spp. were recovered in abundance on the seeds of twenty sunflower varieties sampled [13]. Seed-borne fungi were also found on various parts of sunflower seeds even though no mention of seed coats was made [14,15]. Inner Mongolia region is one of the biggest sunflower planting regions in China, with a planting area of around 0.7 million hectares [16]. Due to the high economic value of sunflower seeds, poor irrigation system, and salty and alkali soil conditions, successive cropping is the main farming system in this region. This causes a gradual increase in pathogen inoculum in the soil, hence, soil-borne diseases such as sunflower verticillium wilt (SVW) and sunflower fusarium wilt (SFW) occurred severely year after year. Other foliar diseases, such as sunflower leaf spot caused by *Alternaria helianthi*, can also be caused by seed-borne inoculum and are known to cause a low yield of sunflower worldwide [17].

Sunflower fusarium wilt, which is caused by several species of Fusarium including *F. oxysporum* and *F. helianthi* [18], is common in many sunflower production fields. The fungal pathogen *Verticillium dahliae* is considered as seed-borne and has been recorded to cause verticillium wilt in over 200 dicotyledonous plant species, including potato and sunflower [19]. This is due to a wide host range of *Verticillium dahliae* that includes lettuce, potato, cauliflower, eggplant, and cotton, thus making it difficult to control it with crop rotation [20,21,22,23].

Hot water treatment is one of the widely used methods to control seed-borne pathogens but due to the hard hull of sunflower seeds, it has not been effective [24]. Flooding is another method used to control seed-borne pathogens like *Fusarium* spp. and *Verticillium* spp., this method is popular in Chinese agriculture especially in the southern part where vegetables are grown [25]. These practices have yielded positive results in most crops but there have not been any significant results with the same methods in sunflower cultivation. This has resulted in the use of chemical fungicide against *Verticillium dahliae* in sunflower seed production. Several fungicides are known to have the potential to control seed-borne fungi such as *F. oxysporum* are Nativo (systemic suspension concentrate containing tebuconazole and trifloxystrobin) and Alliete (water dispersible granule containing alkyl phosphonate) which were tested against fusarium wilt both in vivo and in vitro and was found to be effective in controlling linear and radial colony growth even at lower concentrations [26]. Others include CruiserMaxx (water-based formulation fungicide containing thiamethoxam, difenoconazole, and metalaxyl-M and S-isomer) which is used to treat a wide range of cereals against soil insects and soil-borne pathogens by thoroughly covering the seed surface evenly [27], and stamina fungicide, which is used to treat wheat and barley seeds against soil and seed-borne diseases and contains F-500, triticonazole, and metalaxyl with multiple modes of action [28]. Farmers in most sunflower planting areas in China resort to the use of chemical fungicides mixed with dyes to coat sunflower seeds before planting to protect the seeds from pathogens and pests in the soil. Due to the thick nature of sunflower seed hull, fungicides are unable to penetrate to the seeds. This leaves sunflower seeds contaminated even after pretreatment since only pathogen inoculum on the seeds’ hull surface is reduced but not the seed coats or the seed itself.

Seed-borne diseases such as sunflower verticillium wilt and sunflower fusarium wilt have increasingly affected sunflower production in major farming areas of Inner Mongolia region. Farmers, as well as seed-producing companies, need an efficient way to control seed-borne fungi in sunflower production. To achieve this goal, in this study, we identified various seed-borne fungi morphologically and molecularly on both oilseed and confectionery varieties, and also developed an effective seed pretreatment method to reduce the contamination rate of seeds and prohibit the long-distance transmission of seed-borne fungi via seeds’ transportation.

## 2. Results 

### 2.1. Overview of Contamination Rate and Percentage Range of Contaminated Sunflower Varieties

The sunflower varieties tested had different contamination rates concerning the different fungal colonies recovered growing around seed coats (Table 1). Among the 69 sunflower seeds tested, 11 varieties (16%) were confirmed to be free of fungal contamination, while 58 varieties (84%) were contaminated. The contamination rate ranged from 1% to 22% in the oilseed group and 2% to 90% in the confectionery seed group. A total of 35 varieties had a contamination rate below 11%, representing 60% of the total infected varieties, eight varieties fell between 10% and 30%, and seven varieties between 30% to 50%.

Considering the 52 tested confectionery sunflower varieties, six varieties (JK 601, Chikui 7003, DR 146832, LD 7009, TH 5363, LD 5009) were healthy seeds, making up 10% of the total number of tested varieties, whereas 19 varieties had a contamination rate above 50% which represented 36% of the tested varieties. The confectionery seeds group had 15 varieties with a contamination rate between 10% and 50%. The majority of the tested confectionery varieties were infected by at least one fungi species. The confectionery variety, Jirui 1, recorded the highest contamination rate among all tested varieties, thus 90% (Table 1).

On the contrary, five out of the 17 tested oilseed varieties, New breed 26, LZK 13, LZK 14, Chi Kui CY 101, and Long Kui Za, were free of any fungal contamination representing 29%. The remaining 12 varieties had only *V. dahliae* recovered from their seed coats. The contaminated varieties had nine varieties that had contamination rates between 1% and 11% (Table 2). None of the oilseed varieties tested had a contamination rate above 30%. Among the 12 contaminated oilseed varieties, the highest contamination rate was 22% recorded in the KY 2 variety, whereas the contamination rate of KY 11-23 was the lowest, at 1% (Table 2).

### 2.2. Morphological and Molecular Identification of Fungi Isolated from Seed Coats

**Figure 1:**
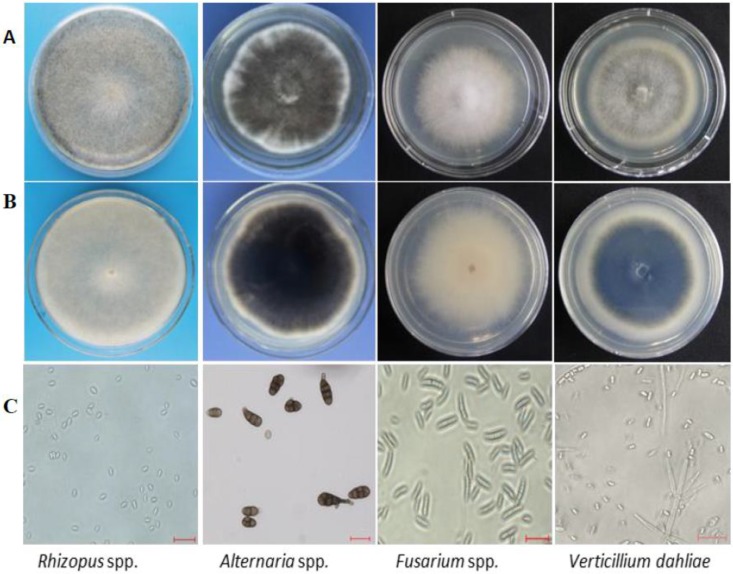
Images of pathogen colonies isolated from tested seed coats. Corresponding colonies’ images after subculturing on PDA (Potato Dextrose Agar) media. (**A**) Front view of culture plate, (**B**) back view of culture plate, (**C**) microscopic view of fungal conidia. Magnification (conidia): 40X, scale bar: 20 µm.

The different fungal colonies recovered from the seed coats of the 58 contaminated varieties were isolated, subcultured, and purified on PDA (Potato Dextrose Agar) media. They were then morphologically identified under the microscope based on their spore and hyphae characteristics. The recovered fungi were generally identified as *V. dahliae*, *Alternaria* spp., *Fusarium* spp., and *Rhizopus* spp. (Figure 1). However, comparing the fungi types recovered from both confectionery and oilseed sunflower varieties, preliminary results suggested that the confectionery varieties were easily contaminated by multiple pathogens, such as *V. dahliae*, *Fusarium* spp., *Alternaria* spp., and *Rhizopus* spp., whereas, *V. dahliae* was the only pathogen isolated from the seed coats of oilseed sunflower varieties.

A total of 46 out of 52 tested confectionery varieties were contaminated by one or more of the fungi identified, the contamination rate was 89%. There were 25 of the tested confectionery varieties that were contaminated by *V. dahliae* only, the other varieties had multiple contaminations, seven varieties were contaminated by both *V. dahliae* and *Alternaria* spp, one variety (Jiarui 3) was contaminated by both *V. dahliae* and *Fusarium* spp., three varieties (Guaner 1, Jishikui 2, Roushui T339) with both *V*. *dahliae* and *Rhizopus* spp., and two varieties (FST 7333, ZH 363) had all four fungi, and *V. dahliae*, *Alternaria* spp., *Fusarium* spp., and *Rhizopus* spp. on the seed coats. Overall, 40% of the confectionery seed lot was contaminated with more than one fungi (Table 1). The highest rate of contamination for *V. dahliae* was recorded in the G1AXR variety, the contamination rate was 57%, for seed contamination by *Rhizopus* spp., ZH 9021 recorded the highest with the contamination rate as 85%, and for *Alternaria* spp., a contamination rate of 90% was recorded in the Jiarui 1 variety. The highest contamination rate of *Fusarium* spp. was 23%, which was recorded in the H16-22 variety. 

**Figure 2:**
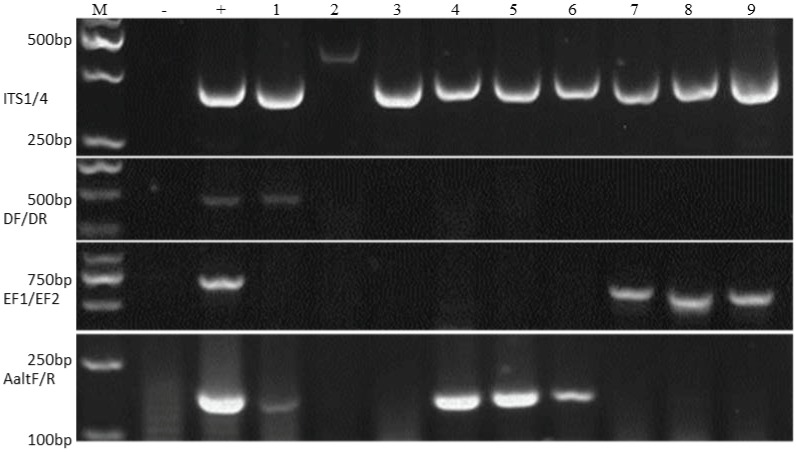
Gel electrophoresis results from PCR (Polymerase Chain Reaction) analysis of subcultured pathogenic colonies on PDA using different primer pairs ITS1/4 (fungi), Df/Dr (*V. dahliae*), EF1/2 (*Fusarium* spp.), and Aalt F/R (*Alternaria* spp.) to identify different pathogen species, respectively. (M: Trans2K DNA marker; − control: double distilled water; + control: *V. dahliae* strain, *Fusarium oxysporum*, and *Alternaria alternata*. 1: *Verticillium dahliae*, 2: *Cladosporium cladosporioides*, 3: Non-Determined, 4–6: *Alternaria alternata*, *Alternaria tenuissima*, *Alternaria helianthiinficiens*, 7–9: *Fusarium oxysporum*, *Fusarium incarnatum*, and *Fusarium proliferatum*).

Morphologically identified fungal species were confirmed using their ITS (Internal Transcribed Regions) region sequence results as query-in data (Figure 2). Extracted DNA from these pure fungal colonies had their ITS regions amplified and sequenced.

An additional fungus, *Cladosporium cladosporioides*, was identified after a nucleotide query-in search using BLAST (Basic Local Alignment Search Tool) (Table 3). This fungus was from PCR sample 2, sample 3 recorded a no-hit during the query-in search.

### 2.3. Fungicide Screening and Optimization of Conditions for Seed Pretreatment

The four broad-spectrum fungicides, carbendazim, triadimefon, pyraclostrobin, and flusilazole were screened against the virulent *Verticillium dahliae* strain, Gn3. Flusilazole was selected after the screening due to its low half-maximal effective concentration value (EC_50_), 78.7 µg/mL. The fungicide producing company’s dilution ratio (10 mL of fungicide: 8000 L of water) was used as a reference to obtain a formulated fungicide volume (*v*/*v*) of 1.25 µg/mL. The formulated fungicide volume was further used to optimize the seed soaking time.

**Figure 3:**
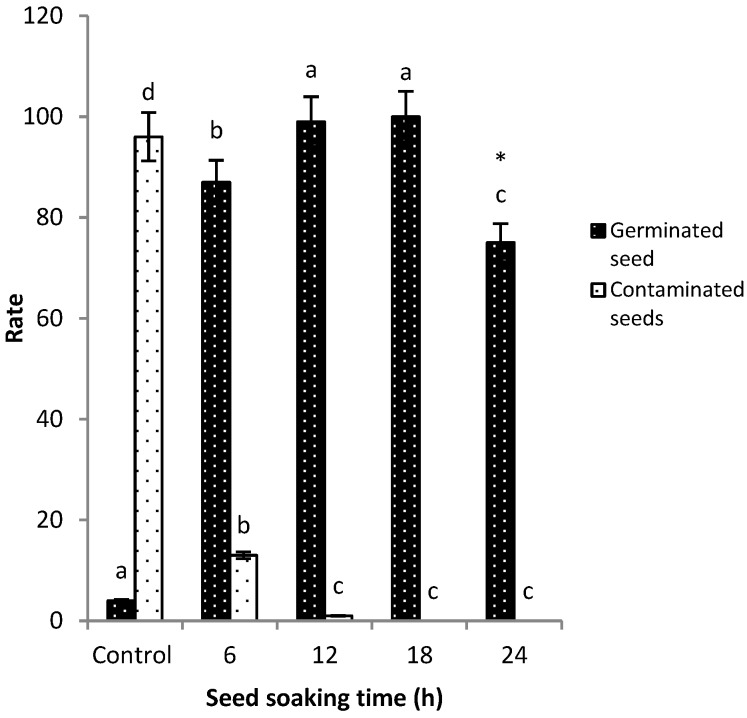
Optimization of seed soaking pretreatment time for flusilazole fungicide. Each treatment had 100 viable LD5009 seeds collected from disease-infected plants from Wuyuan county experimental farm. The 24 h (*) treatment had no contaminated seeds but low seed germination and this might be due to the long exposure of seed to fungicide, using analysis of variance (ANOVA), where *p* ≤ 0.05.

The optimization of seed soaking time was done by soaking seeds in treatments and then placing them on wet filter paper to induce germination. The control treatment was carried out by placing sunflower seeds directly on wet filter paper, and the other three treatments were set up with varying flusilazole fungicide soaking time, as mentioned above in Figure 3. All three treatments had a significant effect on the contamination rate of sprouted seeds as well as seed germination rates. However, the most effective treatment(s) as observed after soaking seeds in the flusilazole solution, was 12 and 18 h, and the seed germination rate was 98% and 100%, respectively (Figure 3). Considering that seeds subjected to 24 h soaking time had no contamination but had less germinated seedlings, this could be due to low seed vigor caused by long exposure of seed to fungicide. Repeated experiments showed a significant difference between seeds soaked for 12 h and those soaked for 18 h in terms of the germination rate. The 12 h soaking time was chosen based on the above results. The optimum fungicide seed pretreatment parameters were set as 12 h seed soaking time in 1.25 µg/mL of the formulated product of flusilazole fungicide solution.

### 2.4. Effects of Flusilazole Pretreatment on Contaminated Sunflower Seeds 

**Figure 4:**
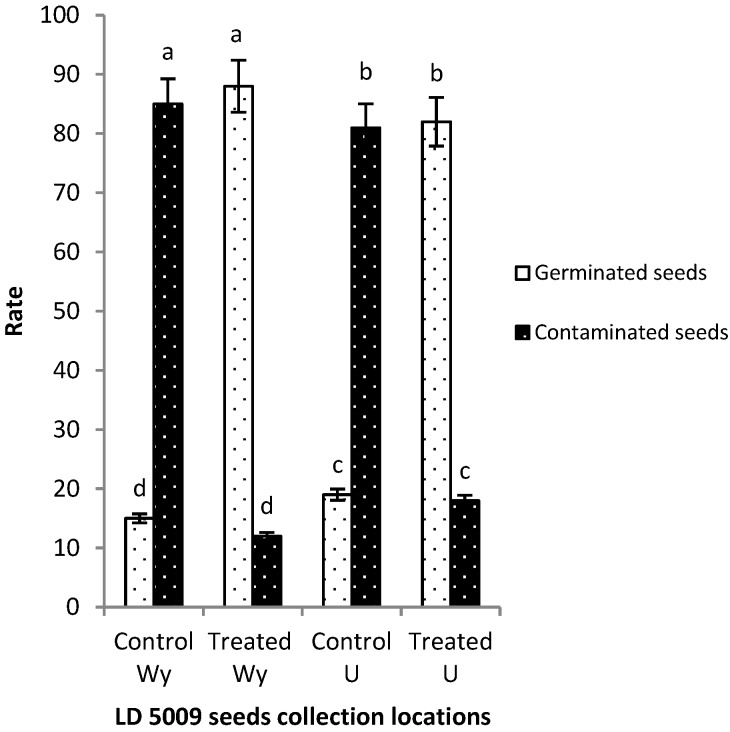
Effect of optimized flusilazole fungicide treatment on sunflower seed hull. Seeds (LD 5009) were collected from different locations. Wy: Wuyuan county, U: Inner Mongolia Agricultural University- Huhhot. Using ANOVA, where *p* ≤ 0.05.

The efficacy of flusilazole pretreatment was evaluated on confectionery sunflower seeds (LD 5009) which were harvested from severely wilted plants from the two experimental farm locations, Wy (Wuyuan county) and U (Inner Mongolia Agricultural University farm). Using the optimum fungicide seed pretreatment parameters selected above, the efficacy of the fungicide was evaluated using emergence as an indication of plant vigor [29]. There were significant differences between the pretreated and untreated seeds in terms of their rate of contamination and seed germination as observed in the two setups. The contamination rate of seeds collected from location Wy was significantly reduced from 85% (control treatment) to 12% (test treatment, with a concentration of 1.25 µg/mL, 12 h dipping) at *p* ≤ 0.05. The rate of germination was significantly increased from 15% (control treatment) to 88% (test treatment) at *p* ≤ 0.05. The seeds collected from location U had a rate of contamination which dropped significantly from 81% (control treatment) to 18% (test treatment), while the germination rate increased from 19% (control treatment) to 82% (test treatment) at *p* ≤ 0.05 (Figure 4).

**Figure 5:**
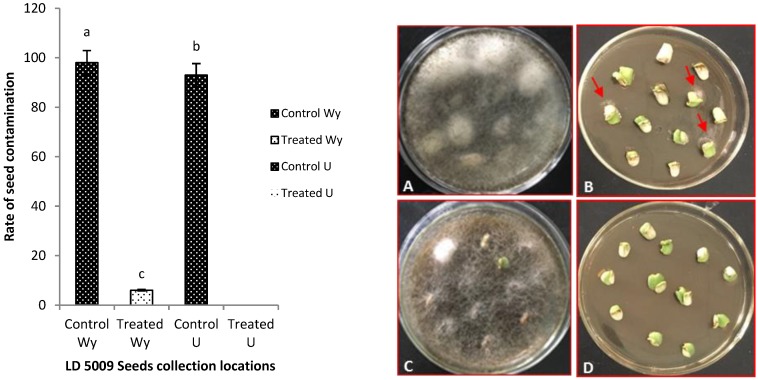
Effects of flusilazole fungicide on controlling fungi on seed hull and seed coat. The red arrows show colonies of fungi around treated seeds coats (less than 10%). Wy: Wuyuan county, U: Inner Mongolia Agricultural University- Huhhot. Using ANOVA, where *p* ≤ 0.05. Labeling: (**A**) Control Wy, (**B**) Treated Wy, (**C**) Control U, (**D**) Treated U.

Both seed emergence and seedling growth were used to evaluate the efficacy of the optimum seed pretreatment as widely accepted in plant response to external factors [30]. After culturing pretreated seeds (seeds with no hull) on PDA medium, the results showed that the seeds’ contamination rate from location Wy decreased significantly from 98% (control treatment) to 6% (test treatment), and that of location U decreased significantly from 93% (control treatment) to 0% (test treatment) at *p* ≤ 0.05, respectively (Figure 5).

## 3. Discussion

Seed-borne sunflower diseases result in a low yield of sunflower seeds production and are difficult to control by using conventional methods. The rapid spread of seed-borne diseases on sunflower farms gave rise to the question of whether the seed coats could be the main tissue for fungi to aggregate. Results from previous research on the contamination process of sunflower verticillium wilt using GFP (green fluorescent protein) -labeled *V. dahliae* showed that the seed coat was one of the main tissues where *V. dahliae* inoculum infect and accumulate [12]. This finding led us to find out the rate of contamination among commercial sunflower varieties with emphasis on their seed coats. The preliminary results of this research confirmed the presence of one or more pathogens on seed coats of 84% of the tested sunflower varieties. This result indicated that sunflower seed coats are the major carriers for long-distance transmission of seed-borne pathogens. *Verticillium dahliae* was detected in almost all the sampled sunflower varieties collected (84%), which suggests that seed-to-seed transmission and long-distance transmission of the fungi is carried out by the seed coat, as seen in spinach production [12]. The presence of multiple fungi on the seed coats supports the fact that seed contamination is the main machinery for the long-distance transmission of seed-borne fungal diseases [7,8,11,31].

### 3.1. Morphological and Molecular Identification of Fungi Isolated from Seed Coats

Based on the comparison of the contamination rate of seeds between confectionery and oilseed sunflower varieties, the confectionery seeds were found to be more susceptible to fungal contamination than the oilseed varieties, since we identified more fungal species from confectionery seeds than from the oilseeds varieties. The G1AXR variety recorded the highest contamination rate (57%) in the confectionery variety group, while KY 2 recorded the highest contamination rate (22%) in the oilseed group. The confectionery group had an average contamination rate of 16%, while the oilseed recorded 7%. This result was backed by a higher susceptibility rate of confectionery seed varieties to *Sclerotinia sclerotiorum* as compared to oilseed varieties [32]. This could be because breeders have been researching oilseed sunflower-resistant varieties for decades, hence, most oilseed varieties grown now are hybrids and have higher disease resistance, as compared to the confectionary type of sunflower [33]. The difference in resistance level could be a result of genetic diversity in confectionery and oilseed sunflower varieties [34]. The genetic structure of the few confectionery varieties that were free of contamination could help breed good resistant varieties.

The BLAST results from the queried fungi sequence confirmed the presence of other fungi on seed coats, apart from *V. dahliae*, which was dominant in both confectionery and oilseed cultivars. *Alternaria* spp. (*A. tenuissima*, *A. alternata*, *A. helianthiinficiens*, *A. longipes*, *A. tamaricis*) and *Fusarium* spp. (*F. oxysporum*, *F. incarnatum*, *F. proliferatum*) were some of the seed-borne fungi found on the sunflower seed coats. The results of this research are parallel to results from Rao [6], where the soil-borne *Fusarium* spp. and *Cladosporium cladosporioides* were also identified on sunflower seed coats. Our findings proved that the increasing disease prevalence and low yield in sunflower planting areas in China are highly related to seed coat contamination which is difficult to treat (Table 1). This can easily increase the transmission of different kinds of diseases from farm to farm, as reported in Reference [35], that seed transmission is a major factor for the transmission and spreading of verticillium and fusarium wilt in China. 

### 3.2. Fungicide Screening, Optimization, and Seed Pretreatment

Sunflower seeds are prone to be contaminated by different pathogens like any other seed, but with the recovery of multiple fungi from sunflower seed coats, an effective reliable seed treatment method must be developed. Fungicide screening was performed to come up with an effective way of pretreating sunflower seeds against soil- and seed-borne diseases. Soaking seeds in formulated flusilazole fungicide for 12 h significantly reduced fungal contamination on the seeds, besides this, seeds’ germination rates were significantly increased. Other works have shown that triazole fungicides can boost seeds’ emergence, as seen in the use of flusilazole fungicide in this study [36]. These findings suggest that pretreatment of seeds with flusilazole can help control fungal contamination in seeds and promote seeds emergence.

## 4. Material and Method

### 4.1. Sample Collection

A total of 69 sunflower varieties, of which 52 were confectionary type and 17 were oilseed type, were collected from different sunflower seed production regions in China in 2017. The detailed information on the varieties tested is listed in Supplementary Table S1. The experiment was performed with 100 randomly selected seeds from each variety and repeated three times. 

Optimization of seed pretreatment with fungicide was done using two lots of the susceptible sunflower variety LD 5009 (from Kaifurui seed company, Beijing) that were collected from two different fields affected with SWV in 2018. One field is located in Wuyuan county, Wy (40°48′24.11″ N 111°42′49.57″ E), one of the biggest sunflower planting areas in Inner Mongolia region, China. The other field is located in the Inner Mongolia Agriculture University farm, U, Hohhot (41°04′43.72″ N 108°03′11.05″ E). The collected seeds were labeled as ‘Wy’ and ‘U’, corresponding to the fields from which they were collected. Seeds were harvested from plants exhibiting severe wilt symptoms (diseased plants).

The virulent *Verticillium dahliae* Gn3 strain was isolated from a diseased LD 5009 sunflower plant in Gannan, Heilongjiang Province of China, and identified using Koch’s postulate as *V. dahliae*. Its GenBank accession number is HQ 441164 [12], this was used as a test strain to determine the efficacy of the fungicides based on its moderate virulent and typical morphology of *V. dahliae*.

### 4.2. Culture Media

Preparation of NP-10 medium and potato dextrose agar (PDA): NP-10 medium is a type of semi-selective medium that consists of two-part components [37]. The first part contains 5 g PGA (polyglycolic acid) (P-3889, Sigma) and 4 g NaOH in 500 mL distilled water, the second part contains 1 g KNO_3_, 1 g KH_2_PO_4_, 0.5 g KCl, 0.5 g MgSO_4_•7H_2_O, and 0.5 mL Tergitol in 500 mL distilled water. The two parts were autoclaved separately at 121 °C for 15 min and then mixed in equal proportion after cooling to an equilibrium temperature of 60 °C. These three antibiotics, 1 mL chloramphenicol (50 g/mL), 1 mL streptomycin sulfate (50 g/mL), and 10 mL chlorotetracycline (50 g/mL), were added before dispensing the medium into sterilized petri dishes.

To prepare, 1 L of PDA, 200 g of potato infusion, 20 g dextrose, and 20 g agar at a final pH of 5.6 ± 2 were added. The potato infusion was made by boiling 200 g of sliced potatoes in 1 L of distilled water for 30 min and the broth was strained through cheesecloth. Distilled water is added to make the final volume of 1 L. The medium was sterilized in an autoclave at a temperature of 121 °C for 25 min (Bacteriological Analytical Manual Media M127). The sterilized media was then dispensed into 9 cm sterile petri dishes.

### 4.3. Determining the Contamination Rate of Sunflower Seed Coats

For each tested variety, 100 viable seeds were randomly selected and cracked open to obtain the seed inside. The seeds were then placed in sterile distilled water for 24 h to soften the seed coats and make them easier to peel off the seed. Surface sterilization of the sunflower seed coats was carried out by placing them in a sterilized sieve and dipping them in 70% ethanol for 1 min, followed by rinsing with sterile double-distilled water for two minutes. Seed coats were then placed on sterilized filter paper in a laminar flow hood to dry. The seed coats were then cultured on freshly prepared NP-10 semi-selective medium (specifically for *V. dahliae*) and PDA. For each petri dish, 10 seed coats were placed on the media and then incubated at room temperature of 24 ± 2 °C for 7 days in a dark chamber. The number of seed coats with fungal colonies growing around was counted after the incubation period. Fungal colonies were transferred to new petri dishes containing PDA media for purification and morphological identification. A sterilized 1000 µL pipette tip (with a diameter of 1 cm) was used to pick a plug of the growing fungal colony around the seed coats and placed on the freshly prepared PDA, which was then incubated at room temperature for 7 days in a dark chamber. A small part of the growing mycelium colony was scraped using a sterile inoculation needle and dropped in a 1.5 mL eppendorf tube containing sterile double-distilled water for morphological identification. An Olympus BX 51 electronic microscope fitted with Qimaging micro-publisher 5 DRTV (for image capturing) was used to repeatedly observe the morphological structures of spores and hyphae of the fungal solution on a glass slide.

The rate of contamination in each variety was calculated using the formula below:

$$Rate\;of\;contamination\;=\frac{Number\;of\;contaminated\;seeds}{Total\;number\;of\;seeds\;tested}$$

### 4.4. Fungi Isolation and Identification

Pure fungal cultures were generated from fungi recovered from confectionary and oilseed seed coats and used for molecular identification. Each petri dish containing purified fungal culture was carefully scrapped with a sterile glass slide cover and placed in a 2 mL eppendorf tube containing ball bearings and then frozen in liquid nitrogen for 2 min, after which it was placed in a Tissue Lyser LT machine (QiaGen Hilden, Germany) for grinding for 2 min at 50 oscillations per second. DNA extraction was carried out following the CTAB (Cetyl Trimethyl Ammonium Bromide) protocol [38]. The successfully extracted DNA samples were used as a PCR template for subsequent fungi species-specific identification based on the amplification of their ITS regions.

The PCR analysis was carried out using primer pairs ITS1/ITS4 [39] to confirm the presence of fungal DNA. Specific primer pairs Df/Dr for *V. dahliae* [40], EF1/EF2 for *Fusarium* spp. [41], and AaltF/AaltR for *Alternaria* spp. [42] were used to identify corresponding species. The primer information is listed in Supplementary Table S2. The amplification was performed with a GenePro Thermal Cycler (Hangzhou Bioer Technology Company Limited, Hangzhou, China, every 25 µL reaction tube contained 1 µL of each primer (10 µM), 0.5 µL Taq DNA polymerase (Tiangen, Beijing, China), 2 µL of dNTP (2.5 mM), 2.5 µL of 10× PCR buffer, 18 µL of distilled water, and 2 µL of template DNA. The PCR was performed at a temperature of 94 °C for 5 min, followed by 35 cycles of 94 °C for 40 s, (ITS1/4) 56 °C for 40 s, 72 °C for 40 s, and finally 72 °C for 10 min for the extension. The time-temperature profile for EF1/EF2 on a GenePro Thermal Cycler (Hangzhou Bioer Technology Company Limited, Hangzhou, China) is 5 min at 95 °C, 30 cycles of 1 min at 95 °C, 75 s at 57 °C, and 1 min at 72 °C, and 10 min at 72 °C, for AaltF/AaltR primers, the procedure was initial denaturation for 3 min at 94 °C followed by 35 cycles at 30 s at 94 °C, and then 60 s at 72 °C. The amplicons were examined electrophoretically on agarose gels stained with ethidium bromide. The amplicons identified from the different primer-specific PCR were then sequenced and used as queried-in search on the GenBank database using the National Center for Biotechnology Information Basic Local Alignment Search Tool (NCBI-BLAST), USA.

### 4.5. Fungicide Screening

Four fungicides (carbendazim, triadimefon, Caprio F-500, and flusilazole) were chosen based on their antimicrobial effects to determine the efficacy of the fungicides (Supplementary Table S3). Carbendazim, which is part of the Methyl Benzimidazole Carbamates (MBC) group of fungicide, targets the β- tubulin gene assembly during mitosis of fungal cells, Caprio F-500 is part of the Quinone outside Inhibitors (QoI) and its target on the cyt b gene, and triadimefon and flusilazole belong to the DeMethylation Inhibitors (DMI) group of fungicides and target on the cyp51 (erg 11) gene [43].

A 1 cm plug of the *V. dahliae* Gn3 strain growing on PDA media was cut out from the edge of the growing colonies using a sterile 1000 µL eppendorf pipette tip and placed on a new PDA media supplemented with the different fungicides. Each of the selected fungicides had a setup of four treatments with different fungicide concentrations, with each treatment having five replicates (Table 4). For each treatment, the calculated amount of formulated fungicide was mixed into 100 mL of freshly prepared PDA and dispensed into 9 cm petri dishes.

The control petri dishes had a 1 cm plug of Gn3 *V. dahliae* subcultured on PDA without any fungicide. The petri dishes were kept in an incubator at a temperature of 24 ± 2 °C for 10 days, after which the inhibitory effect of the fungicide on colony development was calculated by measuring the radius of the colony on each dish and subtracting the initial 1.0 cm plug of Gn3 from it. Efficacy of the fungicides was plotted on the Y-axis and the concentrations on the X-axis respectively, then, the half-maximal effective concentration (EC_50_) was calculated using the linear regression graph formula:

y=mx+c

where ***y*** is 50% of the maximum percentage inhibition and ***m*** and ***c*** are the constants. ***x*** is the concentration(s) listed in Table 4. The fungicide with the least value for its half-maximal effective concentration, ***x*** (EC_50_), was then selected and used in the next stage of the research.

### 4.6. Effect of Flusilazole on Contaminated Sunflower Seed Coats

The selected fungicide seed pretreatment soaking time (12 h) was used to pretreat sunflower seeds collected from severely wilted plants from the two experimental farms Wy and U, in 2018. This experiment involved two setups, setup one involved placing whole seeds (sunflower seeds with seed hulls) on filter paper to initiate germination and the second setup was plating seeds (exposing their seed coats) on PDA media to ascertain the effect of the fungicide seed pretreatment on the possibly contaminated sunflower seed coats.

For these two setups, 400 seeds were randomly selected from seeds harvested from each field and divided into two groups, each one contained 200 seeds. The first group were dipped in a glass jar containing 100 mL of (1.25 µg/mL) flusilazole solution for 12 h. The seeds were allowed to air dry for 24 h and then divided into two equal lots, A and B. The second half of seeds were also divided into two halves to serve as control treatment seeds for the treated seeds (lot A and B). The first lot, A, contained fungicide-treated whole seeds that were placed on wet filter paper kept in a dark room at room temperature. The second lot, B, of treated seeds, had their seed hulls removed as well as the tip of the seeds started to sprout before plating them on PDA media and then kept the plate in an incubator for 10 days at 24 ± 2 °C. The control was also kept under the same conditions. The percentage rate of germination and contamination of sprouts were used as parameters to confirm the efficacy of fungicide treatment. The experiment was repeated three times and the rate of seed contamination was calculated for each treatment after incubating for 10 days. Statistical difference was determined using one-way analysis of variance (ANOVA), this was used to confirm the efficacy of the seed pretreatment protocol developed.

### 4.7. Seed Pretreatment Method

Seed treatment has been one of the main control methods used against pests and diseases in sunflower cultivation. Seed soaking was used as a method for pretreating the seeds collected from the verticillium wilt-infected field. Seeds were soaked in formulated flusilazole fungicide solution with varying intervals (6, 12, 18, and 24 h). The seeds were soaked in 100 mL of formulated flusilazole fungicide. Each treatment had 100 viable seeds soaked in the same concentration of fungicide. The soaked seeds were stirred intermittently to provide uniform exposure of the seed hull surface to the fungicide. Seeds were then dried on sterilized filter papers for 24 h before placing in germination trays lined with wet filter papers to induce germination.

## 5. Conclusions

In this study, we have found that sunflower seed coat is one of the major tissues where soil and seed-borne fungi inocula aggregate, aiding in the rapid and long-distance spread of diseases aside from the use of farm tools in multiple farms. Confectionery sunflower varieties have a higher rate of multiple seed-borne pathogenic contaminations than oilseed varieties, and this supports previous research works [44]. The ability of flusilazole to reduce fungi inoculum on seed hull and seed coats shows the good penetration ability of the fungicide. Therefore, flusilazole is a good seed pretreatment fungicide against soil- and seed-borne fungal contamination in sunflower production. Hence, pretreatment of sunflower seeds by soaking in flusilazole fungicides before planting can help reduce soil- and seed-borne disease incidences on farms and should be encouraged. This could be a favorable method for the control and spread of verticillium wilt caused by *Verticillium dahliae*, which is very difficult to control since its infection pathway is through the vascular system of its host.

## Figures and Tables

**Table 1:** The percentage rate of contamination of tested sunflower confectionery seeds and the various pathogens found.

Sunflower Variety	The Percentage Rate of Pathogenic Contamination (%)				Sunflower Variety	The Percentage Rate of Pathogenic Contamination (%)			
Confectionery Seed Group	*V. Dahliae*	*Rhizopus* Spp.	*Alternaria* Spp.	*Fusarium* Spp.	Confectionery Seed Group	*V. Dahliae*	*Rhizopus* Spp.	*Alternaria* Spp.	*Fusarium* Spp.
G1AXR	57	15	15	0	FST 7331	8	76	0	10
H 16-14	47	0	14	6	Guaner 1	8	70	0	0
H 16-1	42	8	35	0	H 16-22	7	56	0	23
XKS 1618	42	0	0	0	LD 139	7	0	0	0
XKS 1619	37	0	0	0	H 16-20	6	0	0	0
Jishikui 3	35	0	0	0	G2AX12	5	0	0	0
LSK 20	35	0	58	0	Xinhechang 968	5	0	0	0
LSK 21	32	0	0	0	Keyang 4	5	0	0	0
Xiankui 363	32	15	23	0	Fengwo T33	5	0	0	0
Kaifurui 2	28	0	19	0	FST 7333	5	70	10	8
Likuifu 3	27	0	0	0	LJ 316	3	0	0	0
TH 2511	27	52	0	13	Keyang 2	3	0	0	0
Longkui 363	25	0	0	0	Jiarui 3	3	0	0	18
GKS 1601	21	0	0	0	JC 361	3	0	0	0
Gankui 2	20	0	80	0	H 16-24	2	0	0	0
A1X107	20	0	0	0	Chikui 7002	2	0	43	0
Chikui 7001	18	0	22	0	ZH 9021	2	85	0	9
Chikui 7004	18	0	57	0	Jiarui 1	2	0	90	0
ZH 363	17	58	10	6	TF 9041	2	0	0	0
Z1AXR	10	0	0	0	Ruoshui T339	2	84	0	0
A1-ZX 422	10	0	0	0	JK 601	0	0	0	0
Dikui 9233	10	15	63	0	Chikui 7003	0	0	0	0
Z2AXR	9	0	0	0	DR 146832	0	0	0	0
Jishikui 2	8	65	0	0	LD 7009	0	0	0	0
FST 331	8	0	0	0	TH 5363	0	0	0	0
Mengkui 18	8	0	0	0	LD 5009	0	0	0	0

For each variety, 100 viable seeds were cultured and the experiment was repeated three times.

**Table 2:** The percentage rate of contamination of tested sunflower oilseed seeds and the various pathogens found.

Sunflower Variety	The Percentage Rate of Pathogenic Contamination (%)				Sunflower Variety	The Percentage Rate of Pathogenic Contamination (%)			
Oilseed Group	*V. Dahliae*	*Rhizopus* Spp.	*Alternaria* Spp.	*Fusarium* Spp.	Oilseed Group	*V. Dahliae*	*Rhizopus* Spp.	*Alternaria* Spp.	*Fusarium* Spp.
KY 2	22	0	0	0	F 53	3	0	0	0
KY 3	17	0	0	0	S 67	2	0	0	0
ChiKY 11-52	12	0	0	0	KY 11-23	1	0	0	0
France -1	10	0	0	0	New breed 26	0	0	0	0
Longkuiza 2	7	0	0	0	LKZ 13	0	0	0	0
KY 1	3	0	0	0	LKZ 14-4	0	0	0	0
KF 3009	3	0	0	0	Chikui CY 101	0	0	0	0
XKY 1606	3	0	0	0	Longkuiza	0	0	0	0
NKP 218	3	0	0	0					

For each variety, 100 viable seeds were cultured and the experiment was repeated three times.

**Table 3:** Identified pathogen species through BLAST results.

Name of the Genus of Pathogen	Species Type	GenBank Accession Numbers
*Alternaria* spp.	*Alternaria tenuissima*	MN853399
	*Alternaria alternata*	MN853394
	*Alternaria helianthiinficiens*	MN853403
	*Alternaria longipes*	MN853398
	*Alternaria tamaricis*	MN853404
*Fusarium* spp.	*Fusarium oxysporum*	MN853482
	*Fusarium incarnatum*	MN853391
	*Fusarium proliferatum*	MN853400
*Cladiosporium* spp.	*Cladiosporium cladosporioides*	MN853393
*Verticillium* spp.	*Verticillium dahliae*	MN853401
Non-determined	Non-determined	MN853402

These are molecular confirmation of the fungi collected from the sampled seed coats.

**Table 4:** Analytical data of all four fungicides screened and their calculated (Equilibrium Concentration) EC_50_ value.

Fungicide Trade Name	Concentration (µg/mL)	Concentration Per Value (*x*)	Inhibition Rate %	Probability Value (Y)	Virulence Regression Equation	EC_50_ (µg/mL)	R
Carbendazim	5	0.70	72.89%	5.6098	y = −0.8884x + 6.9684	164.3	0.916
	10	1.00	78.29%	5.7824			
	20	1.30	82.76%	5.9463			
	40	1.60	92.63%	6.4466			
Triadimefon	2	0.30	63.51%	5.3451	y = −0.3464x + 5.7089	111.3	0.9104
	3	0.48	65.17%	5.3907			
	7	0.85	69.25%	5.5015			
	13.33	1.12	74.08%	5.6464			
Caprio F-500 (Pyraclostrobin)	0.7	−0.15	32.84%	4.5546	y = −0.4693x + 5.955	131.9	0.9798
	7	0.85	55.54%	5.1383			
	70	1.85	74.36%	5.6557			
	700	2.85	82.76%	5.9464			
Flusilazole	0.013	−1.89	62.04%	5.3055	y = −0.1065x + 5.2019	78.7	0.9937
	0.025	−1.60	63.51%	5.3451			
	0.05	−1.30	64.49%	5.3719			
	0.10	−1.00	65.58%	5.4016			

For each concentration, there were 5 repeats, ***x*** represents the mean measurement of the 5 replicates.

## Supplementary Materials

The following are available online at https://www.mdpi.com/2076-0817/9/1/29/s1, Table S1: Collective data on seeds sampled and their corresponding producing companies, Table S2: Sequence characteristics of primers used in this study, Table S3: Details of formulated fungicides selected for testing.
