# Supplementary material for: Fungicide Treatments to Control Seed-borne Fungi of Sunflower Seeds
Source: Pathogens. 2019 Dec 27;9(1):29. doi: 10.3390/pathogens9010029 (PMC7168664; doi:10.3390/pathogens9010029)
Supplement: Supplementary file 1 [file pathogens-09-00029-s001.pdf]

Table S1. Collective data on seeds sampled and their corresponding producing companies.

Seed variety	Seed group	Seed Producing Company/ Research Institutes	Location	Seed variety	Seeed group	Seed Producing Company	Location
LD 5009	Confectionery	Beijing Kaifurui Company	Alxa League	Jiarui 3	Confectionery	Wuyuan County Jiarui Seed Industry	Bayannur
JK 601	Confectionery	Anhui Huaxia Agricultural Technology Co., Ltd.	Hefei	Mengkui-18	Confectionery	Wuyuan County Wuxing Seed Industry	Bayannur
LJ 316	Confectionery	Hangjinhou Banner, Bayannur City, Inner Mongolia	Bayannur	Dikui 9233	Confectionery	Inner Mongolia Golden Sun Agriculture Co., Ltd.	Chifeng
H 16-1	Confectionery	Baicheng Academy of Agricultural Sciences, Jilin Province	Baicheng	DR 146832	Confectionery	Shaanxi Academy of Agricultural Sciences Crops Institute	Gansu
H 16-20	Confectionery	Baicheng Academy of Agricultural Sciences, Jilin Province	Baicheng	JC 361	Confectionery	Wuchang Huinong Seed Industry, Wuyuan county	Bayannur
H 16-24	Confectionery	Baicheng Academy of Agricultural Sciences, Jilin Province	Baicheng	FS 7331	Confectionery	Inner Mongolia Wuyuan County Harvest Sunflower Seed Industry	Bayannur
H 16-22	Confectionery	Baicheng Academy of Agricultural Sciences, Jilin Province	Baicheng	FS 7333	Confectionery	Inner Mongolia Wuyuan County Harvest Sunflower Seed Industry	Bayannur
H 16-14	Confectionery	Baicheng Academy of Agricultural Sciences, Jilin Province	Baicheng	Xiankui 363	Confectionery	Xiannong International Seed Company	Jiuquan
Gankui 2	Confectionery	Sunflower Research	Qiqihar	LD 139	Confectionery	Ula Special Industry	Bayannur

		Institute of Gannan County, Heilongjiang Province					
Kaifurui 1	Confectionery	Beijing Kaifurui Company	Alxa League	Guaner 1	Confectionery	Liqun Seed Industry	Tongliao
Kaifurui 2	Confectionery	Beijing Kaifurui Company	Alxa League	LD 7009	Confectionery	Ula Special Industry	Bayannur
XKS 1619	Confectionery	Xinjiang Shizei Academy of Agricultural Sciences Crops Institute	Shihezi	TH 2511	Confectionery	Tonghui Seed Industry	Jiuquan
XKS 1618	Confectionery	Xinjiang Shizei Academy of Agricultural Sciences Crops Institute	Shihezi	ZH 363	Confectionery	Gansu Zhenghe Company	Jiuquan
Jishikui 2	Confectionery	Gongzhuling, Siping City, Jilin Province	Gongzhuling	TH 5363	Confectionery	Tonghui Seed Industry	Jiuquan
Jishikui 3	Confectionery	Gongzhuling, Siping City, Jilin Province	Gongzhuling	Likuifu 3	Confectionery	Lanzhou Experimental Station	Lanzhou
Chikui 7003	Confectionery	Chifeng City Academy of Agriculture and Animal Husbandry	Chifeng	GKS 1601	Confectionery	Lanzhou Experimental Station	Lanzhou
Chikui 7001	Confectionery	Chifeng City Academy of Agriculture and Animal Husbandry	Chifeng	Chikui 7002	Confectionery	Chifeng City Academy of Agriculture and Animal Husbandry	Chifeng
Chikui 7004	Confectionery	Chifeng City Academy of Agriculture and Animal Husbandry	Chifeng	LSK 21	Confectionery	Heilongjiang Academy of Agricultural Sciences	Harbin
LSK 20	Confectionery	Heilongjiang Academy of Agricultural Sciences	Harbin	Z1AXR	Confectionery	Heilongjiang Academy of Agricultural Sciences	Harbin
Z2AXR	Confectionery	Heilongjiang Academy of Agricultural Sciences	Harbin	G2AX12	Confectionery	Heilongjiang Academy of Agricultural Sciences	Harbin
Xinhechang 968	Confectionery	Inner Mongolia Jichang Huinong	Bayannur	G1AXR	Confectionery	Heilongjiang Academy of Agricultural Sciences	Harbin

A1-ZX422	Confectionery	Seed Industry Heilongjiang Academy of Agricultural Sciences	Harbin	A1X107	Confectionery	Heilongjiang Academy of Agricultural Sciences	Harbin
Longkui 363	Confectionery	Wuyuan County Yilin Seed Industry	Bayannur	Keying 2	Confectionery	Inner Mongolia Agriculture and Animal Husbandary Academy	Bayannur
ZH 9021	Confectionery	Wuyuan County Taifeng Seed Industry	Bayannur	Keying 4	Confectionery	Inner Mongolia Agriculture and Animal Husbandary Academy	Bayannur
Jiarui 1	Confectionery	Wuyuan County Jiarui Seed Industry	Bayannur	TF 9041	Confectionery	Beijing Kaifurui Company	Alxa League
Ruoshui T339	Confectionery	Wuyuan County Xiangrui Seed Industry	Bayannur	Fengwo T33	Confectionery	Inner Mongolia Wuyuan County Harvest Sunflower Seed Industry	Bayannur
KY 3	Oilseed	Beijing Kaifurui Company	Alxa League	France 1	Oilseed	Agricultural Academy of France	Urumqi
KY 2	Oilseed	Beijing Kaifurui Company	Alxa League	NKP 218	Oilseed	Inner Mongolia Agriculture and Animal Husbandry Academy	Bayannur
KY 1	Oilseed	Beijing Kaifurui Company	Alxa League	F 53	Oilseed	Crop Research Institute, Liaoning Academy of Agricultural Sciences	Liaoning
KF 3009	Oilseed	Beijing Kaifurui Company	Alxa League	KY 11-23	Oilseed	Chifeng city Academy of Agriculture and Animal Husbandry	Chifeng
Xinkui 26	Oilseed	Xinjiang Shihezi Academy of Agricultural Sciences Crops Institute	Shihezi	S 67	Oilseed	Chifeng city Academy of Agriculture and Animal Husbandry	Chifeng

XKY 1606	Oilseed	Xinjiang Shihezi Academy of Agricultural Sciences Crops Institute	Shihezi	KY11-52	Oilseed	Chifeng city Academy of Agriculture and Animal Husbandry	Chifeng
LKZ 13	Oilseed	Heilongjiang Academy of Agricultural Sciences	Harbin	Chi CY 101	Oilseed	Chifeng city Academy of Agriculture and Animal Husbandry	Chifeng
LKZ 14-4	Oilseed	Heilongjiang Academy of Agricultural Sciences	Harbin	Za 2	Oilseed	Crop Research Institute, Gansu Academy of Agricultural Sciences	Lanzhou
				Longkuiza	Oilseed	Institute of Economic Crops, Heilongjiang Academy of Agricultural Sciences	Harbin

From each variety, three lots were taken containing 100 seeds each time. The experiment was repeated three times.

Table S2. Sequence characteristics of primers used in this study.

Primer name	Primer sequence 5' -3'	Tm ˚C
ITS 1	TCCGTAGGTGAACCTGCGG	65.0
ITS 4	TCCTCCGCTTATTGATATGC	58.0
DF	CCGGTCCATCAGTCTCTCTG	54.0
DR	CTGTTGCCGCTTCACTCG	55.0
EF 1	ATGGGTAAGGARGACAAGAC	52.4
EF 2	GGARGTACCAGTSATCATGTT	52.8
AaltF	GTGCCTTCCCCCAAGGTCTCCG	66.1
AaltR	CGGAAACGAGGTGGTTCAGGTC	61.0

These primers were selected based on results from other scientific research work.

Table S3. Details of formulated fungicides selected for testing.

Fungicide trade name/ formulation	Active ingredients	Type of fungicide	FRAC code	Product standard number	Producing company/ Origin
Carbendazim (foliar)	Carbendazol 25% (400g)	Biopesticide	1	HG 3290- 200	Sichuan Guoguang Agrochemical Co. Ltd- China
Triadimefon (foliar)	Triadimefon 15% (50 g)	Systemic fungicide	3	HG 3295- 2001	Sichuan Guoguang Agrochemical Co. Ltd- China
Caprio- F 500 (liquid)	Pyraclostrobin 250 g/L	Strobilurin fungicide	11	Q/DLSK 40- 2012	BASF Chemical Company- Germany
Flusilazole (liquid)	Flusilazole 400 g/L	Organosilicon fungicide	3	Q/ GHNE 45- 2013	DuPont Company- United States of America

These fungicides have abroad spectrum microbial effect and are commonly used for seed dressing.
